# Mass spectrometry‐based analysis of macromolecular complexes of *Staphylococcus aureus* uracil‐DNA glycosylase and its inhibitor reveals specific variations due to naturally occurring mutations

**DOI:** 10.1002/2211-5463.12567

**Published:** 2019-02-09

**Authors:** Veronika Papp‐Kádár, Zoltán Balázs, Károly Vékey, Olivér Ozohanics, Beáta G. Vértessy

**Affiliations:** ^1^ Hungarian Academy of Sciences Research Centre for Natural Sciences Institute of Enzymology Budapest Hungary; ^2^ Department of Applied Biotechnology and Food Science Budapest University of Technology and Economics Budapest Hungary; ^3^ Hungarian Academy of Sciences Research Centre for Natural Sciences Institute of Organic Chemistry Budapest Hungary; ^4^ Department of Medical Biochemistry Semmelweis University Budapest Hungary

**Keywords:** base excision repair, mass spectrometry, protein complex formation, *S. aureus* uracil‐DNA glycosylase inhibitor, SAUDG, SAUGI

## Abstract

The base excision repair pathway plays an important role in correcting damage induced by either physiological or external effects. This repair pathway removes incorrect bases from the DNA. The uracil base is among the most frequently occurring erroneous bases in DNA, and is cut out from the phosphodiester backbone via the catalytic action of uracil‐DNA glycosylase. Uracil excision repair is an evolutionarily highly conserved pathway and can be specifically inhibited by a protein inhibitor of uracil‐DNA glycosylase. Interestingly, both uracil‐DNA glycosylase (*Staphylococcus aureus* uracil‐DNA glycosylase; SAUDG) and its inhibitor (*S. aureus* uracil‐DNA glycosylase inhibitor; SAUGI) are present in the staphylococcal cell. The interaction of these two proteins effectively decreases the efficiency of uracil‐DNA excision repair. The physiological relevance of this complexation has not yet been addressed in detailed; however, numerous mutations have been identified within SAUGI. Here, we investigated whether these mutations drastically perturb the interaction with SAUDG. To perform quantitative analysis of the macromolecular interactions, we applied native mass spectrometry and demonstrated that this is a highly efficient and specific method for determination of dissociation constants. Our results indicate that several naturally occurring mutations of SAUGI do indeed lead to appreciable changes in the dissociation constants for complex formation. However, all of these *K*_d_ values remain in the nanomolar range and therefore the association of these two proteins is preserved. We conclude that complexation is most likely preserved even with the naturally occurring mutant uracil‐DNA glycosylase inhibitor proteins.

**Abbreviations**
AP: apurinic/apyrimidinic
SAUDG: *Staphylococcus aureus* uracil‐DNA glycosylase
SAUGI: *Staphylococcus aureus* uracil‐DNA glycosylase inhibitor
UDG: uracil‐DNA glycosylase
UGI: uracil‐DNA glycosylase inhibitor

The preservation of genome integrity is of key importance for cell viability and faithful transmission of genetic information to subsequent generations. Various damage repair pathways are responsible for efficient and potentially error‐free correction of DNA damage. Among repair pathways, the base excision repair acts to remove base errors due to different chemical reactions, such as oxidation, alkylation and deamination 1, 2.

Base excision repair is initiated by a DNA *N*‐glycosylase enzyme, which is strictly specific for a given modified DNA base. Glycosylase binds to the erroneous base and removes it from DNA, leaving apurinic/apyrimidinic (AP) sites. In the next step, the AP sites are cleaved by AP endonuclease. The repair pathway can follow either a short patch or a long patch route leading towards reconstruction of the original error‐free DNA status 3.

Among the erroneous bases, uracil occurs with a high frequency 4. Uracil may result from the incorporation of dUTP during replication, creating a U:A pair, or the spontaneous deamination of cytosine, creating a premutagenic U:G mispair 5. A recent study indicated that low levels of uracils in genomic DNA of several human cell lines may accumulate in the centromeric regions of chromosomes, although the physiological significance of this finding is yet to be discovered 6. The clearance of dUTP from the cellular pool is therefore of high importance to prevent DNA uracilation. dUTPase enzymes fulfill this role in a highly efficient manner due to their utmost specificity for dUTP paired with a considerable catalytic rate constant 5, 7, 8. If, however, uracil still gets incorporated into DNA or appears from cytosine deamination, another repair enzyme can correct the uracil mistake. Several families of uracil‐DNA glycosylase (UDG) have evolved to cut out uracil from DNA 4, 9, 10. The catalytically most active UDG isoform (encoded by the *ung* gene) is usually present from bacteria to eukaryotes; however, some eukaryote genomes lack the *ung* gene. Based on mutational studies, it is well established that UDG deficiency leads to increased mutational rates 11.

Several inhibitory proteins can modulate catalytic activity of the UDG enzyme. At present, three different uracil‐DNA glycosylase inhibitor proteins have been described in the literature, namely Uracil‐DNA glycosylase inhibitor (UGI) 12, 13, p56 14, 15, 16 and *Staphylococcus aureus* UGI (SAUGI) 17, 18, 19. The amino acid sequences of these inhibitory proteins are strikingly different; however, all of them present a protein surface mimicking the DNA negatively charged double helical structure 20, 21.

UGI is produced by *Bacillus subtilis* PBS1 and PBS2 bacteriophages containing uracil instead of thymine in their genome. The bacteriophages apply UGI to protect their DNA from host cell UDG 12. p56 is produced by *B. subtilis* phi29 phage. Unlike PBS1 and PBS2, this phage does not contain uracil in the genome; however, it has been demonstrated that p56 presents considerable protection for viral DNA replication 14.

The third UGI protein, SAUGI, is encoded by *Staphylococcus aureus* 21. It has been proposed that the gene encoding SAUGI is located in mobile genetic elements of the *S. aureus* genome 22. Different strains of *S. aureus* encode numerous mutated versions of SAUGI 18. While the exact biological role of SAUGI is still unclear, it is highly interesting to note that *S. aureus* also encodes an inhibitory protein for dUTPase, namely Stl 23, 24, 25, 26, 27.

It is therefore apparent that *S. aureus* possesses a complex system for uracil‐DNA metabolism, as detailed in Fig. 1. Both repair enzymes acting against uracil in DNA, dUTPase and UDG, as well as their protein inhibitors, Stl and SAUGI, can be present in the staphylococcal cell, creating intertwined regulatory pathways. It is still unclear how this regulatory potential may be exploited.

**Figure 1:**
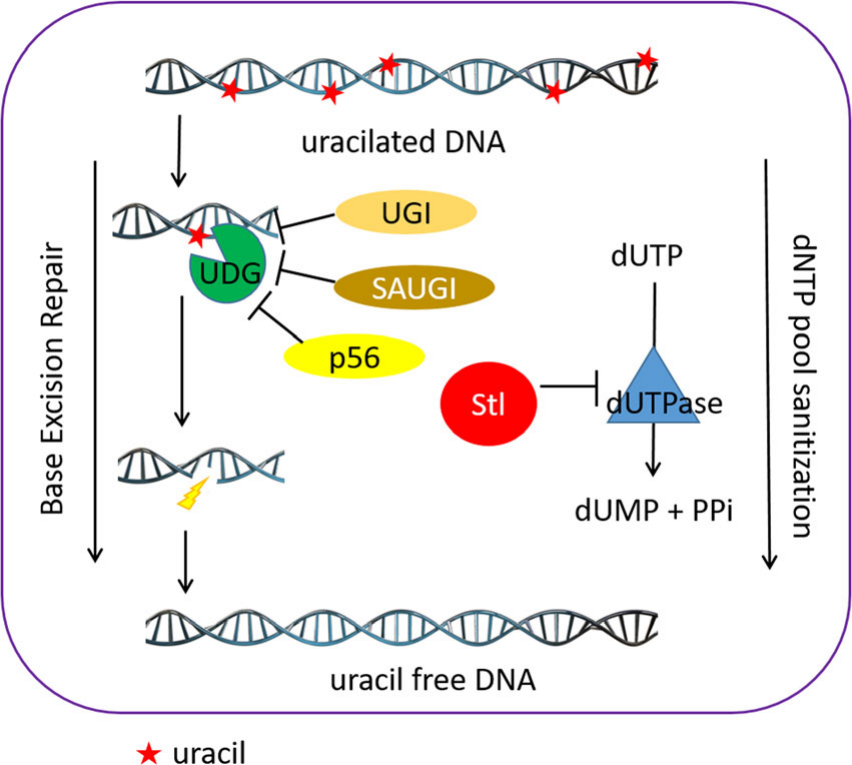
Model of pathways and protein factors collaborating in DNA maintenance. The scheme shows the role of the two main protein enzymes, UDG and dUTPase, in keeping uracil out of DNA. Inhibitor proteins against UDG (UGI, SAUGI and p56, acting in different organisms) and dUTPase (Stl) are also marked on the scheme.

In the present work, we focused on characterization of the interaction between *Staphylococcus aureus* UDG (SAUDG) and SAUGI, using mass spectrometry as a sophisticated state‐of‐the art method. Our aim was to investigate whether naturally occurring mutations within the SAUGI sequence may have major consequences for complex formation. We therefore constructed several mutant SAUGI proteins and analyzed their binding to SAUDG exploiting native mass spectrometry.

## Materials and methods

### *In silico* Blast search and alignments

For homologous sequences of SAUGI proteins, the NCBI Blast search was performed using the wild‐type SAUGI sequence (Uniprot code: Q936H5). The search was performed using translated nucleotide query (blastx), and the protein sequences database was non‐redundant, in the *S. aureus* (taxid 1280) organism. The alignment sequences similarity was higher than 90%, which was adjusted manually.

### Cloning and mutagenesis

SAUDG and SAUGI vectors were from H.‐C. Wang (Taipei Medical University) 17. A His6 tag was inserted into the SAUGI encoding vector.

The SAUGI mutant constructs were engineered by site‐directed mutagenesis using the QuickChange method (Agilent, Santa Clara, CA, USA). Primers used for mutagenesis (Table 1) were synthesized by (Eurofins Genomics GmbH, Ebersberg, Germany). Constructs were verified by DNA sequencing at Eurofins MWG GmbH.

**Table 1:** Primers for constructing E24H, H87E, D59Y, M89K and I50T point mutations

SAUGI^E24H^	F‐Primer	5′ – cctaccaaaggatgaaaagtgg**cat**tgtgaatctatcgaggaaatcg – 3′
	R‐Primer	5′ – cgatttcctcgatagattcaca**atg**ccacttttcatcctttggtagg – 3′
SAUGI^H87N^	F‐Primer	5′ – tcggctatatcgatgaaaat**aac**gatatggatttcttatacctacac – 3′
	R‐Primer	5′ – gtgtaggtataagaaatccatatc**gtt**attttcatcgatatagccga – 3′
SAUGI^D59Y^	F‐Primer	5′ – cctatacctactactct**tat**acacttcacgaaag – 3′
	R‐Primer	5′ – ctttcgtgaagtgtata**aga**gtagtaggtatagg – 3′
SAUGI^M89K^	F‐Primer	5′ – cgatgaaaatcacgat**aag**gatttcttatacctac – 3′
	R‐Primer	5′ – gtaggtataagaaatc**ctt**atcgtgattttcatcg – 3′
SAUGI^I50T^	F‐Primer	5′ – ggggcactcagtaataaa**aca**cttcaaacctatacctac – 3′
	R‐Primer	5′ – gtaggtataggtttgaag**tgt**tttattactgagtgcccc – 3′

The bases, coding the altered amino acids are highlighted in bold letters.

### SAUGI and SAUDG protein expression and purification

Protein expression and purification were performed as described previously 19. In brief, expression was performed in *E. coli* Rosetta BL21 (DE3) PlysS cells (Novagen, EMD Biosciences, Inc., Merck KGaA, Darmstadt, Germany) at 16 °C. Cells were harvested by centrifugation (4 °C, 4000 ***g***, 20 min), and extracted by sonication. Cell supernatants were used for purification of the proteins on an Ni‐NTA column. Elution of SAUDG, SAUGI^WT^ and mutant SAUGI constructs was done using elution buffer (137 mm NaCl, 2.7 mm KCl, 10 mm Na_2_HPO_4_, 2 mm KH_2_PO_4_, pH 7.5).

### Mass spectrometry

Mass spectrometry measurement was performed on a (Waters, International Equipment Trading Ltd, Mundelein, IL, USA) Q‐Tof Premier mass spectrometer equipped with an electrospray source. The instrument parameters were set up as follows: electrospray ionization capillary voltage 2.8 kV, source temperature 90 °C, desolvation temperature 160 °C, cone gas flow 25 L·h^−1^, desolvation gas flow 600 L·h^−1^, cone voltage 60 V, extraction cone 5.0 V, ion guide 3.5 V, ion guide gas flow 10 mL·min^−1^. Samples were introduced by direct injection with a flow rate of 10 μL·min^−1^. Mass spectra were obtained under native conditions in the 800–7000 *m*/*z* range; the ions were generated from aqueous 50 mm NH_4_HCO_3_ buffer solution (pH 7.8) containing the protein at 2 μm SAUDG and 0.5–2 μm SAUGI concentration.

### Data analysis

To determine the dissociation constant, each sample was mixed from the same SAUDG stock solution. For each inhibitor protein tested, seven SAUDG/inhibitor ratios were measured, while keeping the SAUDG concentration 2 μm. The measured data were analyzed by summing all spectra during the 5 min measurement and taking into account the three most intense peaks belonging to the SAUDG protein and three to the complex. After calculating the ratio of the protein complex peak area to the SAUDG peak area, we used a modified formula from Daniel *et al*. 28 to fit the data. Formula parameters were fitted using the Levenberg–Marquardt algorithm.

(1) $$\begin{array}{cc} & \frac{I(P*L)}{I(P)}=\\ & \frac{1}{2}*\left(-1-\frac{{\left[P\right]}_{0}}{K}+\frac{{\left[L\right]}_{0}}{K}+\sqrt{4\frac{{\left[L\right]}_{0}}{K}+{\left(\frac{{\left[L\right]}_{0}}{K}-\frac{{\left[P\right]}_{0}}{K}-1\right)}^{2}}\right)*c, \end{array}$$

where *I*(P*L) and *I* (*P*) are the measured signals for the complex and the free SAUDG, respectively. [*P*]_0_ and [*L*]_0_ are the initial protein concentrations and *K* is the association constant. The dissociation constant can be calculated as 1/*K*. As demonstrated previously, the microchannel plate detector has three to five times lower signal for a two times increase in mass, depending on instrument parameters, and therefore a constant, *c*, was introduced.

## Results and Discussion

Our aim was to apply native mass spectrometry to determine dissociation constants characterizing the interaction between SAUDG and naturally occurring mutant variants of SAUGI. It has been already recognized that native mass spectrometry is a relevant and suitable technique to investigate proteins in their native conformation 26, 29. However, data characterizing dissociation constants of macromolecular complexes obtained by mass spectrometry are still rare in the literature 30.

In order to select putatively relevant mutant variants of SAUGI, we performed sequence alignments of all SAUGI sequences present in public databases (27 sequences). Since the physiological function of SAUGI, to our current knowledge, is inhibition of SAUDG, we focused on those peptide segments present in the SAUGI protein that are known to be involved in complex formation with SAUDG (the three‐dimensional structure of the SAUGI–SAUDG complex has been published: PDB ID 3WDG) 17.

**Figure 2:**
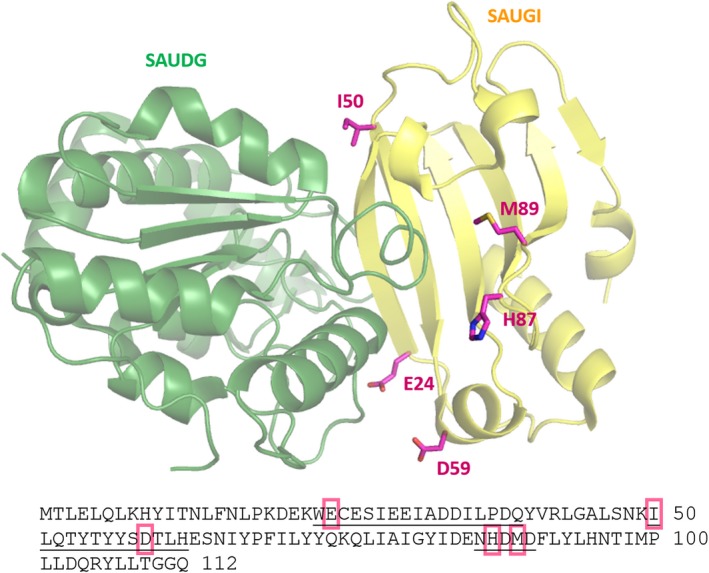
Structural representation of SAUDG:SAUGI complexes (visualized with pymol Graphics System, DeLano Scientific, San Carlos, CA, USA). Three‐dimensional structural model of the complex formed by SAUDG (green cartoon) and SAUGI (yellow cartoon) (PDB: 3WDG). Residues within the SAUGI protein, selected for further study are depicted in stick model with purple carbons and atomic coloring. Bottom panel presents the amino acid sequence of SAUGI. In the sequence, amino acids participating in the formation of the SAUDG:SAUGI complex surface are underlined, and positions selected for further study are highlighted in purple.

Figure 2 presents the SAUGI sequences wherein we have indicated the peptide segments involved in the complex formation. In addition, residues where mutant variants were selected for further study are highlighted. Figure 2 also shows the position of the selected residues within the three‐dimensional structure of the complex. The rationale for selecting individual mutant variants considered the change in the character of the residue upon mutation: those mutations were considered where major variation occurred in hydrophobicity or charged/polar character.

**Figure 3:**
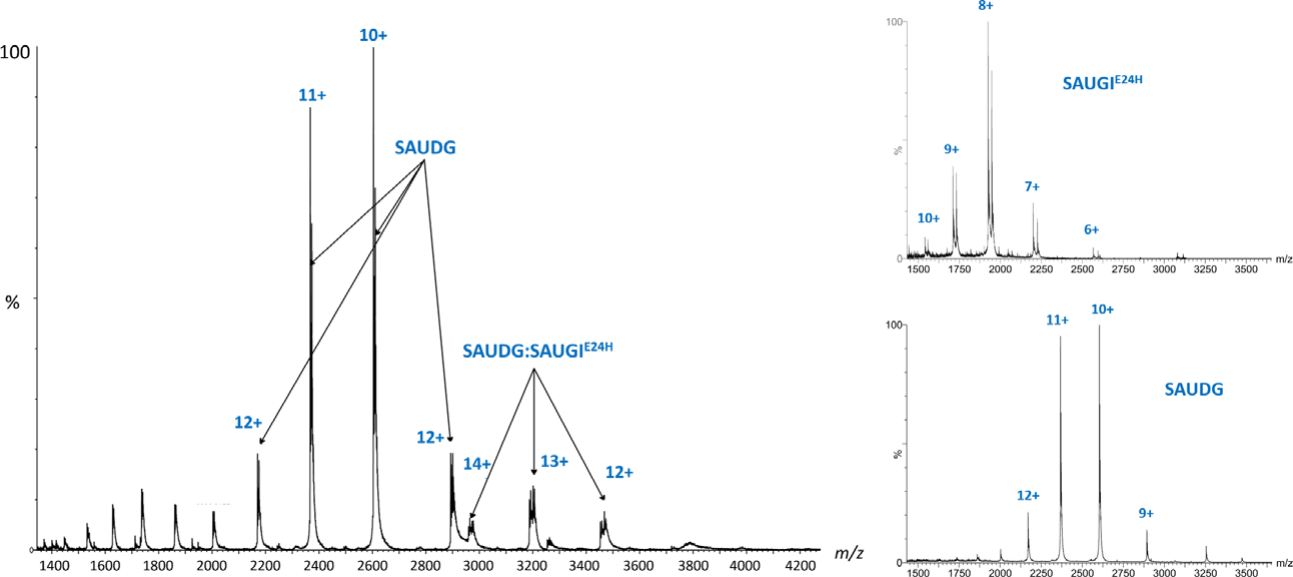
Mass spectrometric analysis of the protein complex, where the concentration of SAUDG and SAUGI^E^ ^24H^ was 2 and 1.25 μm, respectively.

We have measured the complex formation of SAUDG and five naturally occurring mutants (SAUGI^E24H^, SAUGI^I50T^, SAUGI^D59Y^, SAUGI^H87N^, SAIUGI^M89K^) using mass spectrometry. Preliminary experiments indicated that the 0.5–2 μm concentration range for the protein complex result is optimal to obtain well‐characterized spectrum signals. Figure 3 shows a representative mass spectrum of the SAUDG:SAUGI^E24H^ complex.

**Figure 4:**
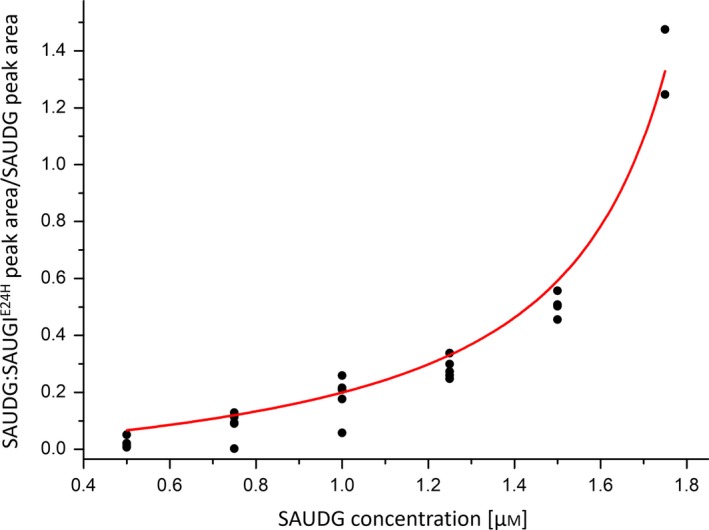
Determination of the dissociation constant of the SAUDG:SAUGI^E^ ^24H^ complex. Data are plotted according to Materials and methods. The fitted curve (red line) is also shown.

In the spectrum, peaks assigned to SAUDG and the protein complex are marked. No peaks associated with free SAUGI^E24H^ can be observed. The ratio of the complex and free SAUDG signals is a relevant measure of the complex formation. As described in Materials and methods, the dissociation constant was calculated based on this ratio, determined using several measurements with different concentrations of the SAUGI protein, while the concentration of SAUDG was kept constant. Figure 4 presents the complex to free protein ratio as a function of the SAUGI^E24H^ concentration. For the other mutants, the titration curves can be found in the Supporting information (Fig. S1).

The dissociation constants obtained for the complexes of SAUDG with wild‐type and mutant SAUGI constructs are listed in Table 2. We note that among the different mutant variants, one SAUGI construct possesses a somewhat stronger interaction with SAUDG, while the other mutations either weaken the interaction or do not perturb it.

**Table 2:** Dissociation constants of SAUGI E24H, H87E, D59Y, M89K and I50T mutations

	*K*_d_ (nm)
SAUGI^WT^	14.4 ± 1.5
SAUGI^E24H^	1.7 ± 1.4
SAUGI^H87N^	69.4 ± 5.5
SAUGI^D59Y^	96.3 ± 8.0
SAUGI^M89K^	38.3 ± 1.0
SAUGI^I50T^	14.1 ± 0.2

Previously the complex formation between wild‐type SAUGI and the SAUGI^E24H^ mutant has been partially investigated by microscale thermophoresis and isothermal titration microcalorimetry 19. With regard to the dissociation constant determined for the SAUDG:SAUGI^WT^ complex, it is of interest to note that our currently determined value obtained by mass spectrometry (14.4 ± 1.5 nm) compares more favorably to the dissociation constant of the same complex determined by surface plasmon resonance (1.2 nm) 17, as compared with the data obtained by isothermal titration microcalorimetry (131 ± 31 nm) 19. It has been already observed that the lengthy microcalorimetry technique is not optimal for proteins that are sensitive to stirring, temperature and buffer/salt conditions. Since a microcalorimetry titration takes about 2 h to be completed, it can be used only for proteins that can withstand these conditions without significant conformation changes. Both SAUDG and SAUGI proteins showed a tendency to precipitate in the microcalorimeter. The surface plasmon resonance technique also presented significant difficulties in the determination of the dissociation phase, due to the exceptionally strong complexation between SAUDG and SAUGI. Considering these difficulties, a different approach was sought, and mass spectrometry proved to be applicable.

**Figure 5:**
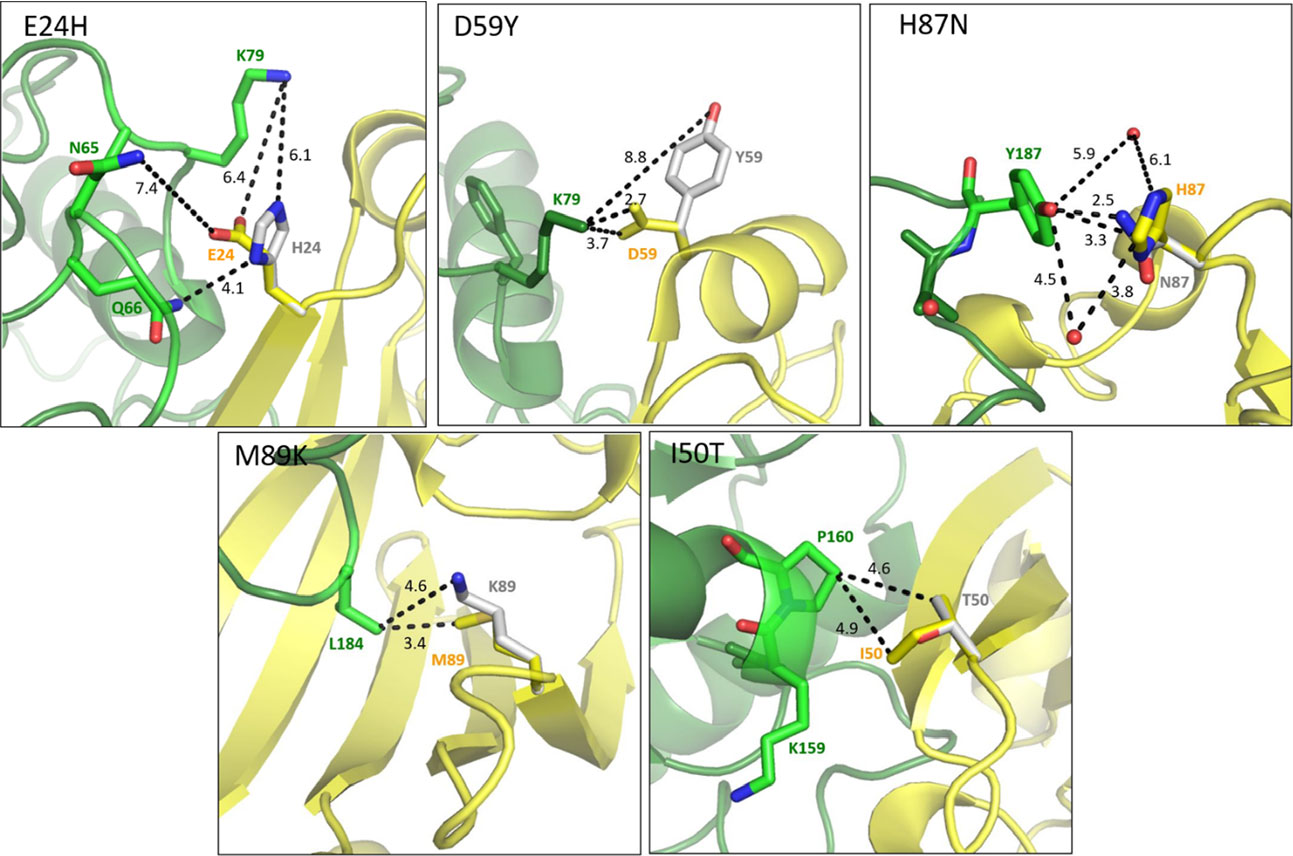
Three‐dimensional structural model of the complex formed by SAUDG (green cartoon) and SAUGI (yellow cartoon) (PDB: 3WDG). Shown are the proposed conformation of several mutated residues of SAUGI (SAUGI^E^ ^24H^, SAUGI^D^ ^59Y^, SAUGI^H^ ^87N^, SAIUGI^M^ ^89K^ and SAUGI^I^ ^50T^) at the SAUDG: SAUGI interaction surface. Residues involved in interactions with the selected SAUGI mutations are shown in stick model, colored according to atomic coloring (carbons are in yellow for the wild‐type and in light gray for the mutants). Only the most probable mutant amino acid conformer generated by the pymol Mutagenesis tool is depicted. Dashed lines with numbers indicate atomic distances in ångströms.

As shown in Table 2, we have observed that among the investigated SAUGI mutants there is one in which the strength of complex formation is highly increased. In this specific case (E24H mutation), a glutamic acid residue has been replaced by a histidine. In the complex structure of SAUDG:SAUGI, this glutamic acid residue does not participate in any strong interaction (cf. distances shown on Fig. 5, respective panel). However, the histidine residue in the mutant variant may form polar contact with a glutamine residue (Q66) of SAUDG.

In three further mutations, the dissociation constant is considerably increased (SAUGI^H87N^, SAUGI^D59Y^ and SAUGI^M89K^). In all these cases, inspection of the three‐dimensional structure provides relevant considerations, in agreement with the weakening of the complexation (Fig. 5). For the SAUGI^H87N^ mutant, it is well observable that the histidine residue in the wild‐type SAUGI participates in the aromatic interaction with tyrosine 187 (Y187) of SAUDG. The asparagine mutation fails to perform this interaction, hence weakening complex formation.

Considering the SAUGI^D59Y^ mutant, it is to be noted that in the wild‐type SAUGI, the aspartic acid residue 59 (D59) forms a charged interaction with the amino group of lysine 79 (K79) of SAUDG. This strong charged interaction is lost when aspartate 59 is mutated to tyrosine.

For another mutant (SAUGI^M89K^), a methionine residue in the wild‐type SAUGI is accommodated in a hydrophobic pocket of SAUDG. Substitution of this methionine for a charged lysine residue interferes with this interaction thereby weakening complex formation.

Finally, the SAUGI^I50T^ mutant exhibits a similar *K*_d_ to wild‐type SAUGI. This is in good agreement with the fact that the isoleucine residue in the wild‐type protein does not participate in any strong interactions with SAUDG.

In conclusion, our experimental data indicate that several naturally occurring mutant variants of SAUGI still allow considerably strong complex formation with SAUDG. Even in the strongest perturbation (observed for the SAUGI^D59Y^ variant), the dissociation constant with SAUDG is rather low in the submicromolar range. We propose that under physiological conditions, the observed subtle variations of the strength of complexation between SAUDG and the SAUGI variants may not have strongly disturbing effects. Our data therefore argue that complexation between SAUGI and SAUDG is evolutionarily conserved and only those mutant variants of SAUGI are preserved that still constitute a relevant interaction network with SAUDG.

## Conflict of interest

The authors declare no conflict of interest.

## Author contributions

VP‐K: experimental set‐up planning, *in silico* blast and alignment, cloning and mutagenesis, protein expression and purification, sample preparation for mass spectrometry, paper writing. ZB: protein expression and purification. KV: mass spectrometry data analysis. OO: mass spectrometric measurements, and data analysis, paper writing. BGV: research group leader, supervisor, experimental set‐up planning, data analysis, paper writing.

## Supporting information


**Fig. S1.** Determination of the dissociation constant of the SAUDG:SAUGI variant complexes, according to Materials and methods. The obtained data set (black points), and the fitted curve (red line) are shown in the case of SAUGI^WT^ (A), SAUGI^I50T^ (B), SAUGI^D59Y^ (C), SAUGI^H87N^ (D), and SAIUGI^M89K^ (E).
