# Supplementary material for: Mass spectrometry‐based analysis of macromolecular complexes of Staphylococcus aureus uracil‐DNA glycosylase and its inhibitor reveals specific variations due to naturally occurring mutations
Source: FEBS Open Bio. 2019 Feb 9;9(3):420–7. doi: 10.1002/2211-5463.12567 (PMC6396141; doi:10.1002/2211-5463.12567)
Supplement: Supplementary file 1 — Fig. S1. Determination of the dissociation constant of the SAUDG:SAUGI variant complexes, according to Materials and methods. The obtained data set (black points), and the fitted curve (red line) are shown in the case of SAUGIWT (A), SAUGII50T (B), SAUGID59Y (C), SAUGIH87N (D), and SAIUGIM89K (E). [file FEB4-9-420-s001.pdf]

Supplementary Material

Supplementary Figure S1

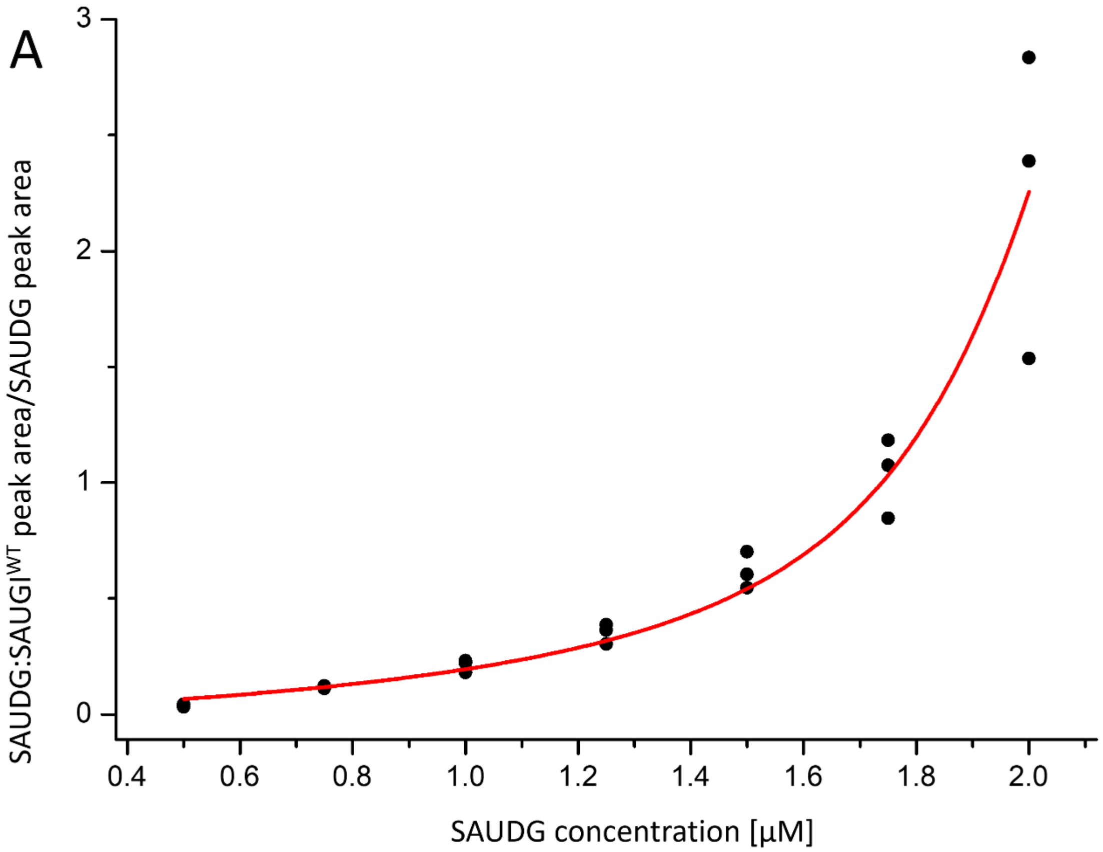

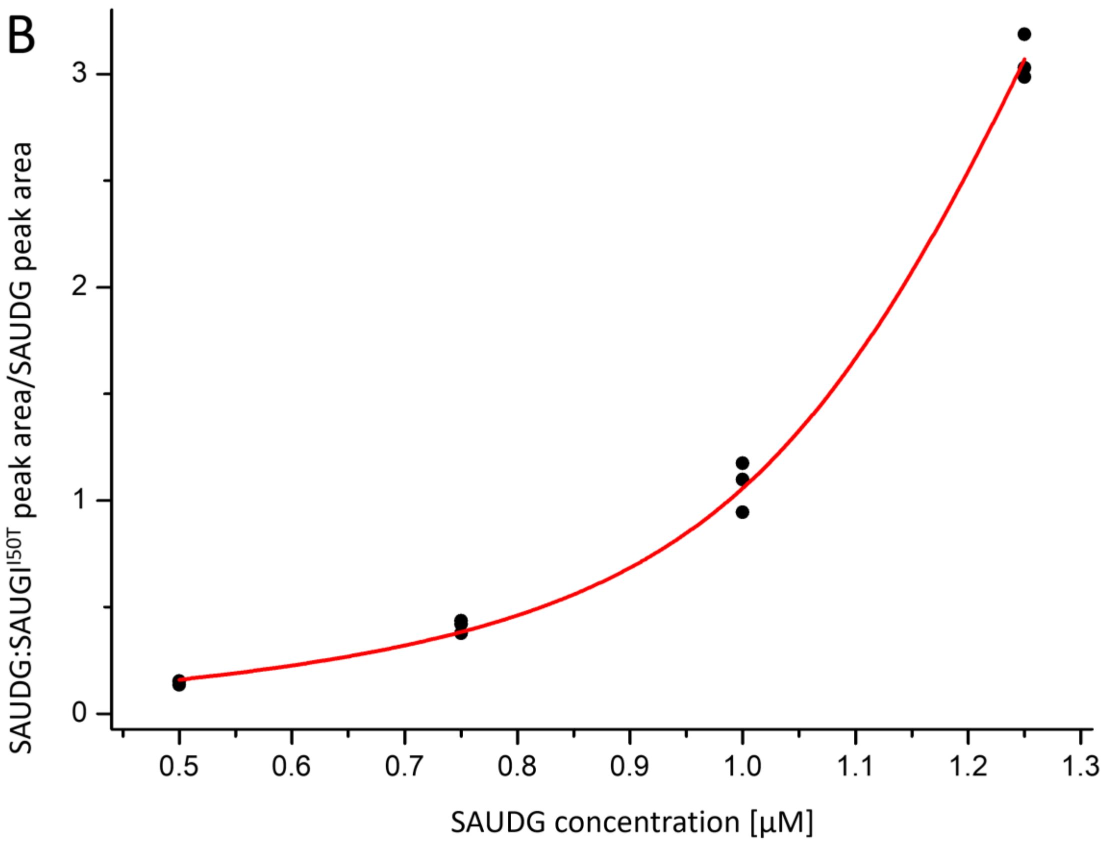

B
SAUDG:SAUGI[I50T] peak area/SAUDG peak area
0
1
2
3
0.5
0.6
0.7
0.8
0.9
1.0
1.1
1.2
1.3
SAUDG concentration [μM]

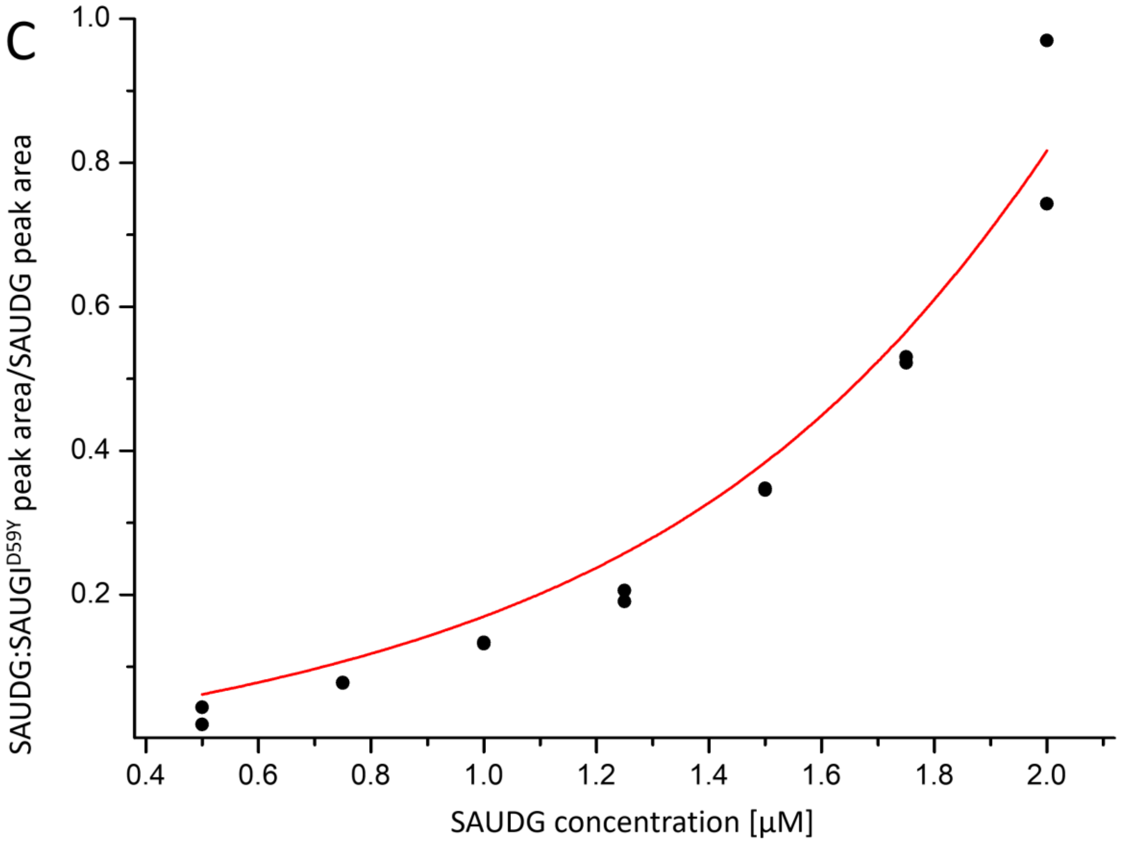

C
SAUDG:SAUGI^{D59Y} peak area/SAUDG peak area
1.0
0.8
0.6
0.4
0.2
0.4
0.6
0.8
1.0
1.2
1.4
1.6
1.8
2.0
SAUDG concentration [μM]

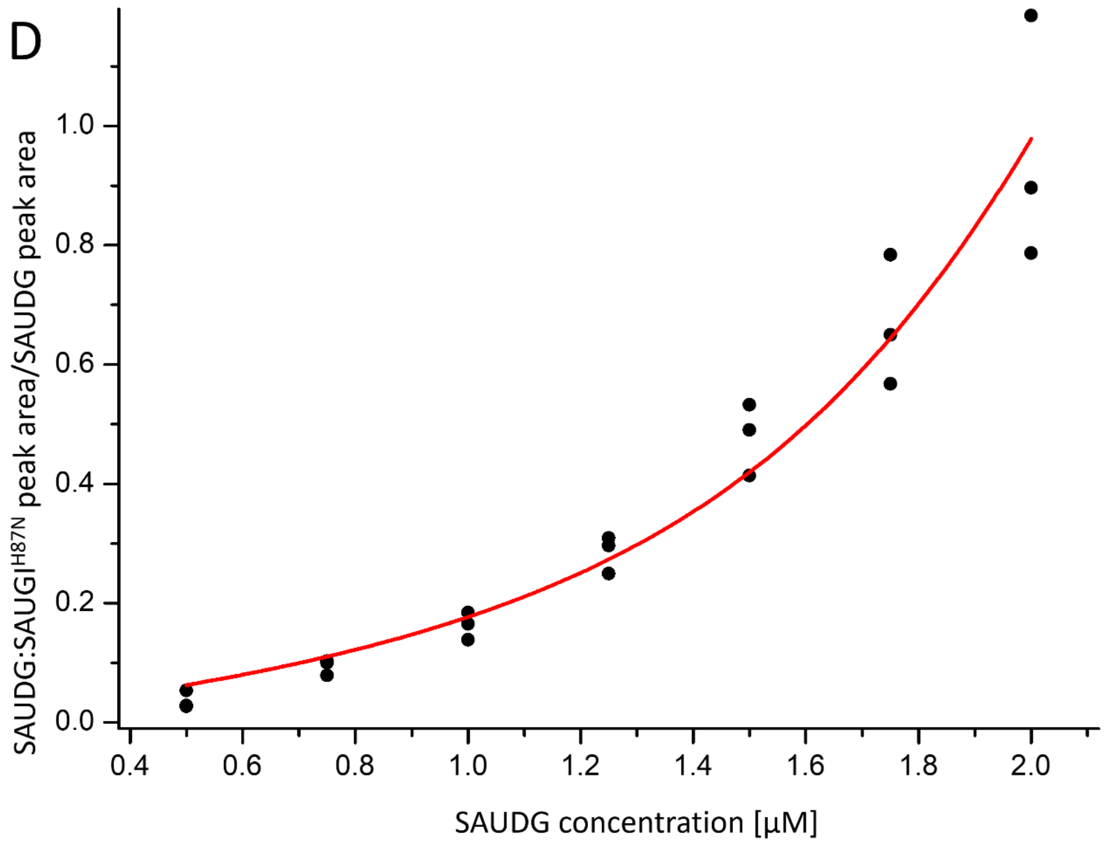
D
SAUDG:SAUGI^{H87N} peak area/SAUDG peak area
0.0
0.2
0.4
0.6
0.8
1.0
0.4
0.6
0.8
1.0
1.2
1.4
1.6
1.8
2.0
SAUDG concentration [μM]

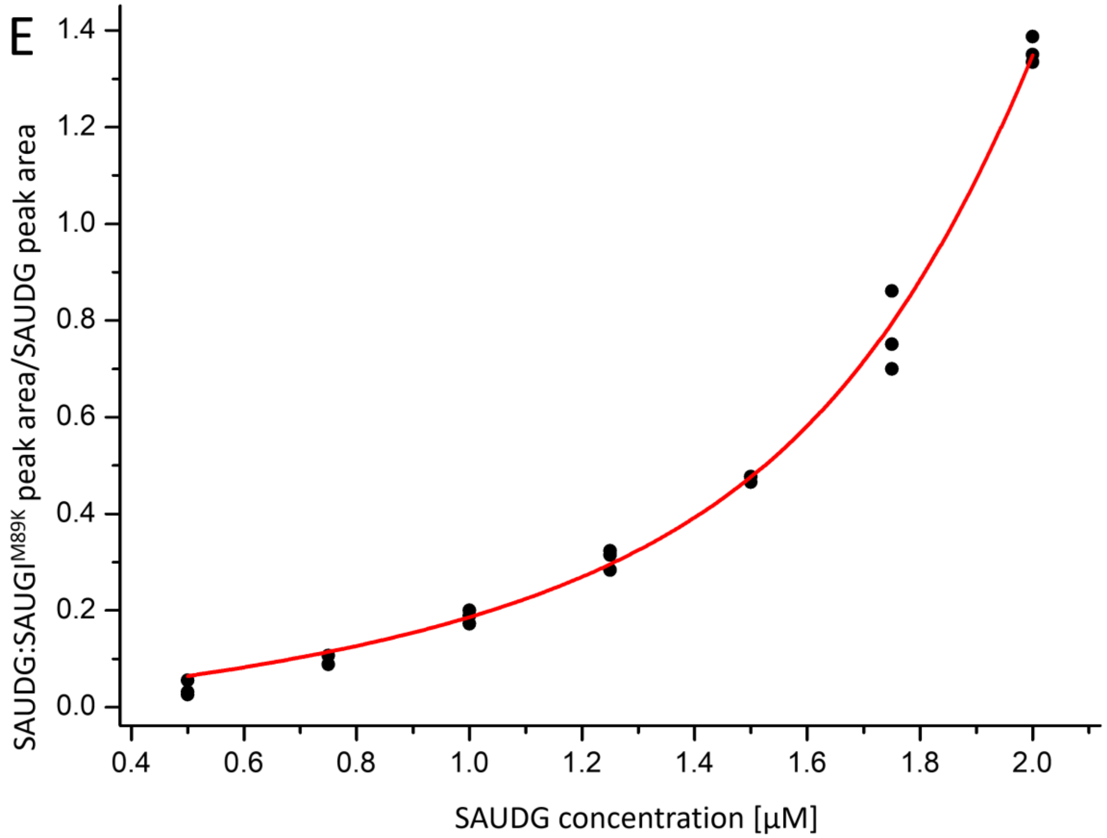

Supplementary Figure 1. Determination of the dissociation constant of the SAUDG:SAUGI variants complexes, according to Materials and Methods. The obtained data set (black points), and the fitted curve (red line) are shown in the case of SAUGI^{WT} (panel A), SAUGI^{I50T} (panel B), SAUGI^{D59Y} (panel C), SAUGI^{H87N} (panel D), SAIUGI^{M89K} (panel E), respectively.
